# Supplementary material for: Defining Metaniches in the Oral Cavity According to Their Microbial Composition and Cytokine Profile
Source: Int J Mol Sci. 2020 Nov 3;21(21):8218. doi: 10.3390/ijms21218218 (PMC7663680; doi:10.3390/ijms21218218)
Supplement: Supplementary file 1 [file ijms-21-08218-s001.pdf]

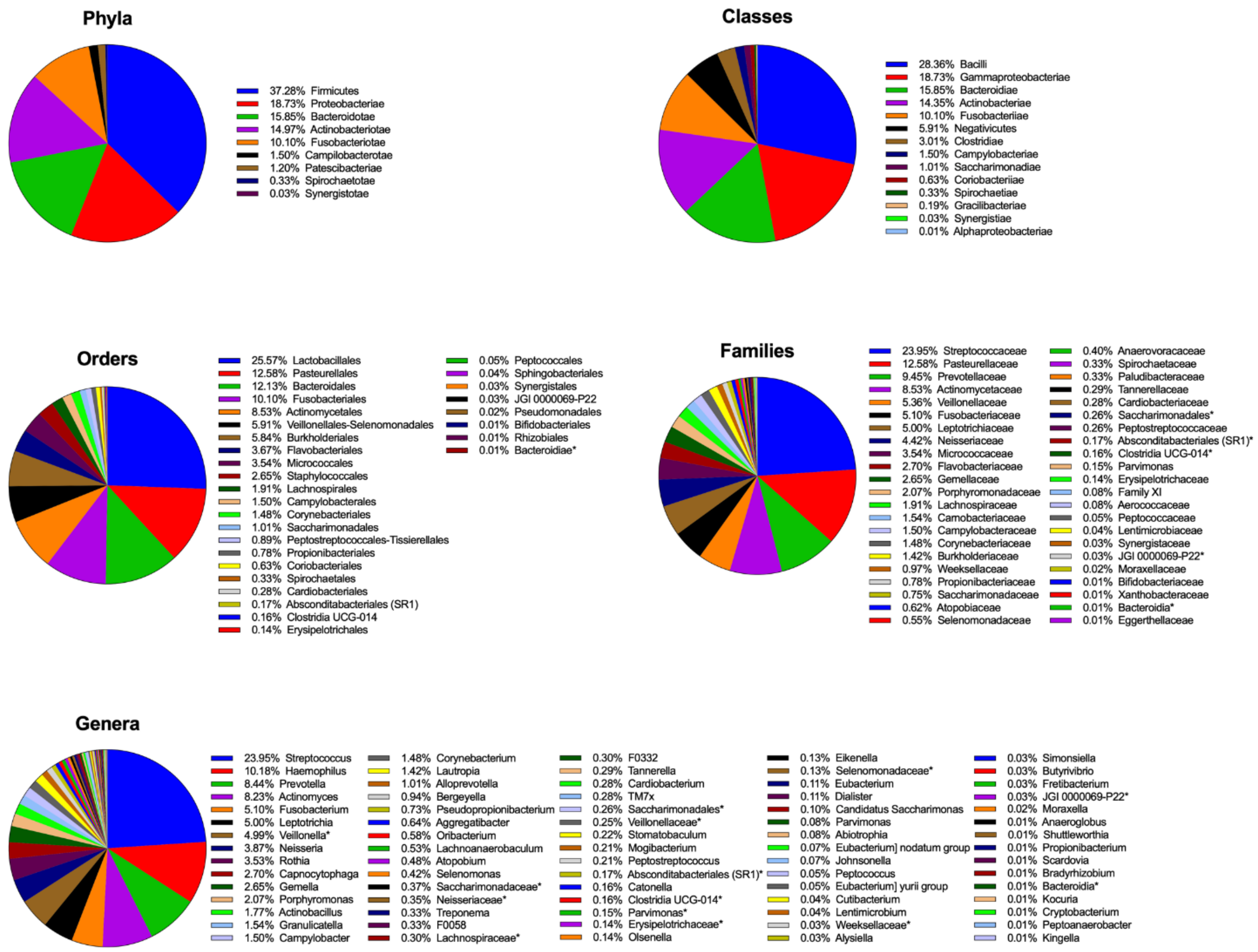

Figure S1: Oral microbiota composition as detected by sequencing of 16S rRNA genes in order of ranked-based classification on phylum level, class level, order level, family level and genus level. * indicates unnamed specification.

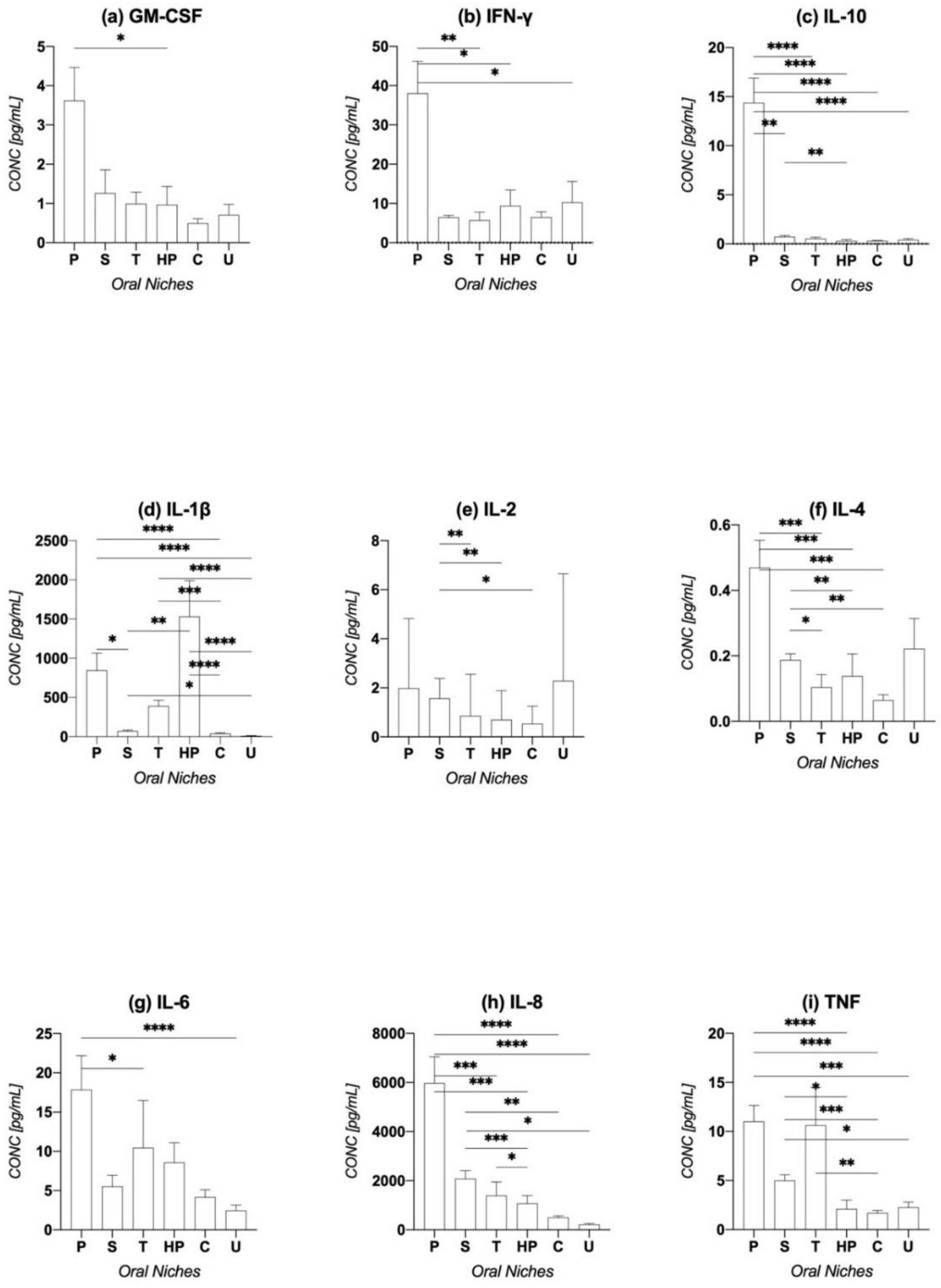

Figure S2. Histograms representing the concentrations of (a) GM-CSF, (b) IFN-γ, (c) IL- 10, (d) IL-1ß, (e) IL-2, (f) IL-4, (g) IL-6, (h) IL-8, (i) TNF in defined oral niches. P, supragingival plaque; S, saliva; T, tongue; HP, hard palate; C, cheek; U, sublingual area. The data is presented as mean and standard error of mean (SEM). N=20, * 0.01 ≤ p < 0.05, **0.001 ≤ p < 0.01, *** 0.0001 ≤ p < 0.001, ****p< 0.0001.

Table S1. Clinical parameters of the study population are given. Number, #; Periodontal probing depth, PPD; Bleeding-on-Probing-Index, BOP; Approximal-Plaque-Index, API.

**Subject #**	**Teeth**	**PPD**	**BOP**	**API**	**Oral Hygiene**		
					**Toothbrush**	**Floss**	**Prophylaxis**
001	28	1.6	0%	14%	powered	regularly	regularly
002	28	1.4	0.6%	18%	powered	occasionally	regularly
003	28	1.6	0%	11%	powered	occasionally	regularly
004	28	1.6	0%	18%	powered	occasionally	regularly
005	32	1.7	0.5%	7%	powered	regularly	regularly
006	28	2.1	2.4%	18%	powered	occasionally	regularly
007	28	1.6	2.4%	14%	powered	regularly	regularly
008	32	2.1	1%	14%	powered	regularly	regularly
009	28	2.3	0%	14%	powered	regularly	regularly
010	32	1.5	0%	14%	powered	occasionally	regularly
011	28	1.7	1.2%	11%	powered	occasionally	regularly
012	32	1.7	0%	18%	powered	regularly	regularly
013	28	1.8	0%	14%	powered	occasionally	regularly
014	30	1.4	0%	11%	powered	regularly	regularly
015	28	1.8	0%	7%	powered	regularly	regularly
016	26	2.1	1.3%	7%	powered	regularly	regularly
017	28	1.9	0.6%	14%	powered	occasionally	regularly
018	28	1.9	1.2%	11%	powered	regularly	regularly
019	28	2.4	1.2%	18%	powered	regularly	regularly
020	31	2.1	1.6%	7%	powered	occasionally	regularly
